# A Case of Acral Papulovesicular Rash Associated With COVID-19

**DOI:** 10.7759/cureus.55438

**Published:** 2024-03-03

**Authors:** Serap Maden

**Affiliations:** 1 Dermatology, Near East University, Faculty of Medicine, Nicosia, CYP

**Keywords:** covid-19, rash, acral, papulovesicular, cutaneous manifestations

## Abstract

COVID-19 is a global pandemic that started in 2020. During the pandemic, patients have presented with various cutaneous manifestations of COVID-19 infections. Currently, COVID-19 infections are still present worldwide, although to a lesser extent. This case report describes a 21-year-old male patient who presented with papulovesicles on his hands and lateral aspects of his ankles for three days. The patient also experienced respiratory symptoms for eight days and tested positive for COVID-19. It is important to have an understanding of the skin manifestations associated with COVID-19, as this can aid in the diagnosis and comprehension of the condition.

## Introduction

The coronavirus known as severe acute respiratory syndrome coronavirus 2 (SARS-CoV-2), responsible for the COVID-19 infection, caused a global pandemic in 2020 as recognized by the World Health Organization [1]. Skin manifestations associated with COVID-19 include a variety of clinical presentations, such as morbilliform maculopapular eruption, vesicular (pseudo-varicella) rash, urticarial rash or urticaria, chilblain-like lesions, and acro-ischemia [2]. Several case reports and small case series describe cutaneous lesions in the hands and feet, identified as acral cutaneous eruptions, which have been observed in association with COVID-19 infections [3]. Understanding, defining, and categorizing skin findings in patients with COVID-19 can help clinicians suspect early-stage SARS-CoV-2 infections in patients with few symptoms. This objective approach can aid in prompt diagnosis and treatment. Here, a case of papulovesicular rash on the acral regions that was thought to have developed in association with COVID-19 is presented.

## Case presentation

**Figure 1:**
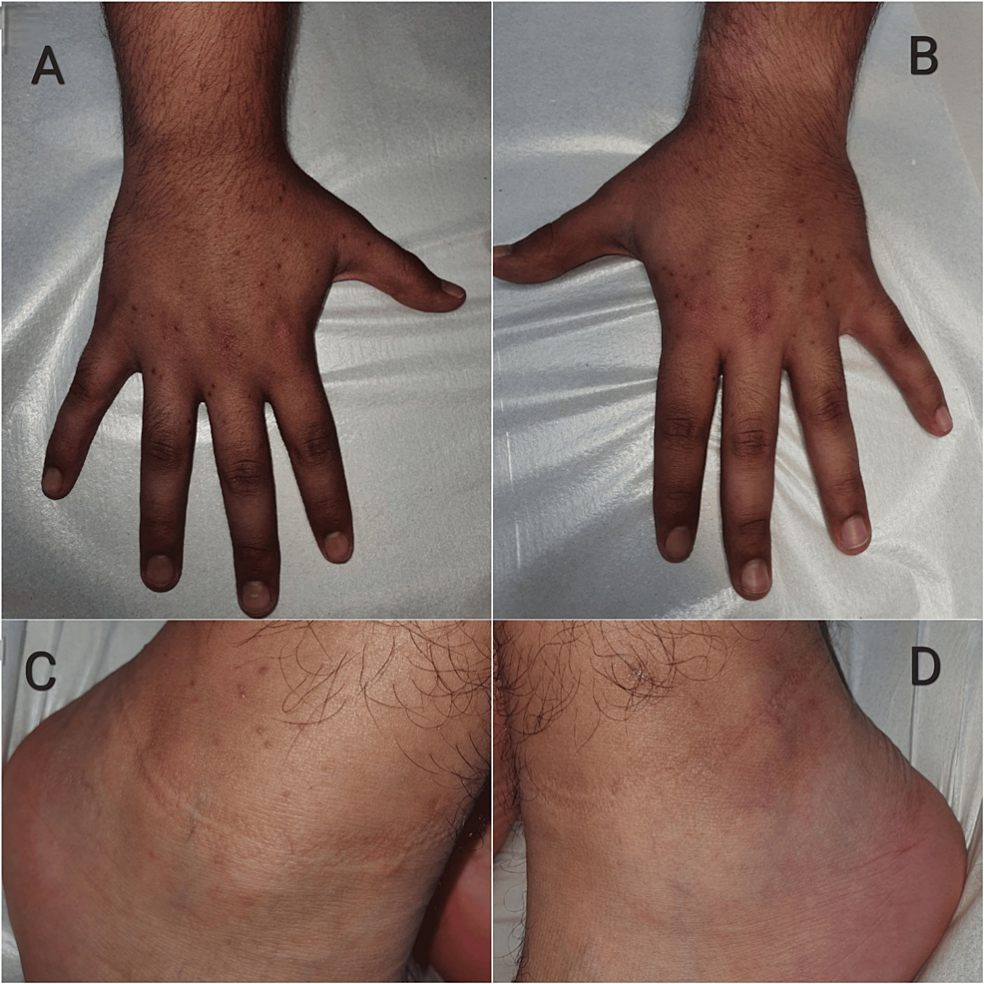
Monomorphic, symmetrical, scattered, erythematous papulovesicules on the extensor surfaces of the hands and lateral aspects of the ankles. The distribution of lesions on the dorsal surface of the hands (A, B). There were fewer lesions on the lateral aspects of the ankles compared to those detected on the hands (C, D).

**Figure 2:**
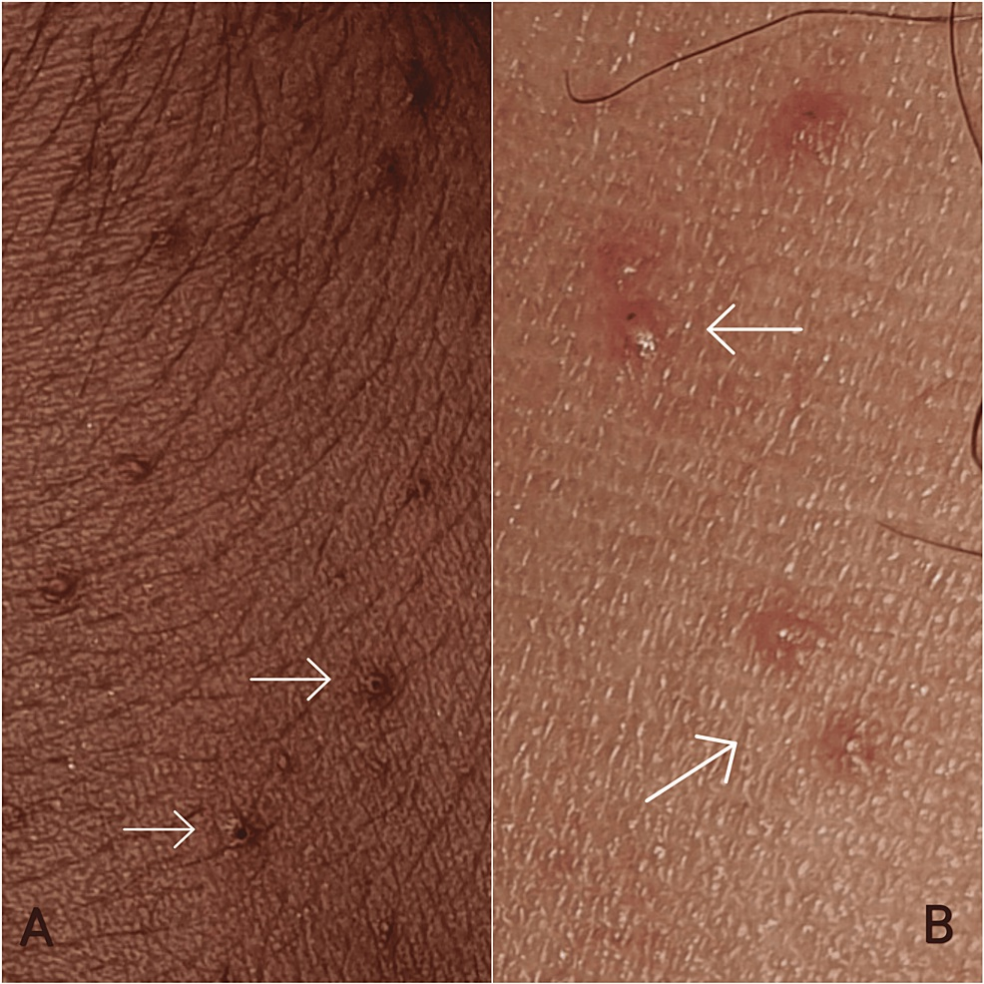
A closer view of the papulovesicles. Lesions on the dorsum of the hands (A) and on the lateral aspects of the ankles (B).

A 21-year-old male patient presented to our clinic as an outpatient due to experiencing itching symptoms and the appearance of bumpy areas on his hands for three days. He had been experiencing respiratory symptoms, including a dry cough and runny nose, for over a week but did not take any medication. He did not report any additional medical conditions or history of medication use. Dermatologic examination revealed scattered, monomorphic, erythematous papulovesicules, 2-3 mm in diameter, on the extensor surfaces of the hands, with a symmetrical distribution. In comparison to the papulovesicules observed on the hands, there were fewer lesions on the lateral aspects of the ankles (Figures 1, 2).

Based on the patient's eight-day history of respiratory symptoms and three-day history of rash, we recommend COVID-19 testing. SARS-CoV-2 real-time polymerase chain reaction was conducted using nasolabial and oropharyngeal swab samples obtained from the patient, resulting in the positive detection of SARS-CoV-2 RNA. Routine blood tests, including complete blood count and assessments of the liver and kidney function and C-reactive protein, did not reveal any abnormalities. The patient reported not receiving any COVID-19 vaccinations for protection prior to today. The Tzanck smear was not performed due to the patient's normal immune status and the clinical presentation of the lesions. The appearance and distribution of the lesions did not suggest a Herpesviridae infection in the clinical differential diagnosis due to their scattered and monomorphic nature. A skin biopsy was planned for the lesions, but the patient declined the procedure. Based on the clinical presentation of respiratory symptoms, cutaneous findings, and laboratory results, the patient was diagnosed with acral papulovesicular rash due to a COVID-19 infection. The skin lesions resolved spontaneously within 10 days without requiring any treatment. No additional systemic or cutaneous manifestations were reported.

## Discussion

Skin manifestations related to COVID-19 infections have been extensively documented in the existing literature [2]. Polymorphous or atypical rashes, such as erythema multiforme-like eruptions, leukocytoclastic vasculitis, pityriasis rosea and pityriasis rosea-like rashes, and COVID nose, have also been reported in association with COVID-19 infections [2].In Marzano et al.’s study, involving 22 patients with papulovesicular eruptions associated with COVID-19 infections, the authors differentiated those of “true” varicella and identified them as a varicella-like exanthem. In the case series, no variations in the papulovesicular presentation were observed. The study population had a median age of 60 years, with 72.7% (n = 16/22) being male. The trunk was always involved and, in some cases, associated with the extremities (n = 4; 18.2%). Six patients (27.3%) presented with diffuse papulovesicular exanthema, while the majority (n = 16; 72.7%) presented with scattered papulovesicular exanthema. The skin lesions appeared on average three days after the onset of systemic symptoms and disappeared within eight days without leaving any scarring [4].

Similar to the cases in the study, our case presented as a male patient with scattered and monomorphic lesions. However, unlike the study, the patient was young and had no trunk involvement. Furthermore, lesions appeared five days after the onset of respiratory symptoms and resolved within 10 days. Moreover, the lesions in our case primarily clustered in the distal extremities, which can be classified as acral. Acral skin manifestations resulting from a COVID-19 infection consist of various types of lesions, such as acral papulovesicular eruption, acral urticarial lesion, acral non-inflammatory purpura and skin necrosis, acro-ischemia, acral vasculitis, chilblain-like lesion (also known as "COVID toe"), acral erythema multiforme-like lesion, acral lesions associated with multisystem inflammatory syndrome in children, acral peeling lesions, and red half-moon nail sign [3].

The diagnosis of papulovesicular exanthem associated with COVID-19 is debated by some authorities, as it may be confused with varicella zoster virus or herpes simplex virus. To confirm the diagnosis, a Tzanck smear can be performed [5,6]. On the other hand, Genovese et al. suggested that while seeking evidence for *Herpes viridae *family members may be ideal, clinical diagnosis is generally reliable. The authors mentioned that it is important to consider the possibility of herpes viruses acting as a superinfection in patients with dysfunctional immune responses associated with COVID-19 [7]. In our case, the symmetrical distribution of the papulovesicles distinguished these lesions from those caused by herpes zoster or localized herpes virus infection. The focus on the monomorphic pattern and the limited involvement of the lesions on the extremities have diverted our attention from the possibility of varicella infection. Therefore, we did not perform a Tzanck smear based on the clinical presentation of the lesions and the patient's normal immune status.

Based on the localization and characteristics of the lesions, our case is consistent with acral papulovesicular eruption. This case may be indicative of Gianotti-Crosti syndrome due to the location of the lesions on the extremities and their papulovesicular characteristics, as well as their self-limiting behavior. However, a definitive diagnosis cannot be established due to the absence of lesions on the face, palms, and soles, as well as the lack of histopathological findings.

Immunopathological mechanisms have been implicated in COVID-19 rashes, which are characterized by papules and papulovesicules. For instance, papular eruptions due to COVID-19 infections may present as immune complexes or delayed hypersensitivity reactions [8]. In addition, acral papulovesicular lesions are suggested to be a result of immune system hyperactivation and the direct cytopathic effect of SARS-CoV-2 on endothelial skin vessels [9].

In the literature, a case of papulovesicular eruption with onset in the acral region was presented following the first dose of the Pfizer-BioNTech COVID-19 vaccine in an 18-year-old male patient. This eruption progressed to a generalized papulovesicular eruption after the second dosage of the vaccine [10]. Nevertheless, in our case, we observed a self-limiting papulovesicular exanthem on the acral region, which did not become generalized. The variation in lesion severity observed in both cases can be attributed to the immune system's response to the antigenic origin, but it is important to note that this comparison is between vaccination and infection. A recent study found no correlation between COVID-19 vaccinations and flare-ups of psoriasis, which is a chronic inflammatory skin disease. The study also found no correlation between exposome parameters, such as age, gender, time of onset, and disease flare-ups, in COVID-19-vaccinated patients. Furthermore, the study suggests that vaccination during the summer months may provide protection against psoriasis flare-ups [11]. Based on these results, COVID-19 vaccination may also provide protection for individuals with chronic skin conditions.

For COVID-19-related papulovesicular exanthem, there are currently no standardized treatments available. In the literature, there are case reports presenting papulovesicular rash due to COVID-19 infections that healed without any treatment and presented as self-healing skin exanthemas. An eight-year-old girl with COVID-19 presented with 40 erythematous papulovesicles on the trunk, which were described as varicella-like exanthema. The patient's skin lesions subsided without any therapy within seven days [12]. Another report describes a 10-month-old boy with a COVID-19 infection who presented with papulovesicles on his cheeks, upper and lower extremities, and buttocks. The case was diagnosed as Gianotti-Crosti syndrome, and no additional treatment was recommended to the patient because the rash is self-resolving [13]. It could be noted that the papulovesicular rash is self-healing within a short period of time. Therefore, a “wait-and-see” strategy may be recommended for these cases [7]. The lesions in our case resolved spontaneously, without necessitating any treatment, serving as an illustration of self-healing. Although a "wait-and-see" approach may be taken in cases of papulovesicular exanthemas, topical corticosteroids have been found to be beneficial in some reported cases. For instance, in a 23-year-old male diagnosed with Gianotti-Crosti syndrome due to COVID-19, the papulovesicles resolved within weeks with the use of topical corticosteroids, specifically betamethasone, applied once a day [14]. The 25-year-old female patient presented with a Gianotti-Crosti-like eruption accompanied by a maculopapular exanthema. A one-week course of methylprednisolone cream was administered as a topical corticosteroid, resulting in a progressive resolution of the lesions in 11-18 days [15]. The availability of standardized treatments for skin lesions associated with COVID-19 infection is crucial for controlling skin diseases. In addition, dermatologists can effectively prevent complications, such as lesion generalization or secondary infections, by administering appropriate treatment to patients.

## Conclusions

This report attributes an acral papulovesicular rash to the COVID-19 viral infection. The viral etiology and immune mechanism can cause skin manifestations in patients with COVID-19 infection. The appearance of papulovesicular exanthem may serve as a useful indicator of COVID-19 diagnosis in paucisymptomatic patients, prompting further investigation by clinicians. Furthermore, understanding skin findings in patients with respiratory symptoms can help clinicians detect early-stage COVID-19 infection and prevent complications. Categorizing the skin findings can also aid in determining the etiology and ruling out differential diagnoses. However, while a "wait-and-see" approach may be appropriate for certain cases of papulovesicular exanthema, it would be advantageous to establish standardized treatments for skin lesions related to COVID-19 infection to better manage the condition.
